# Commensal Bacteria: Not Just Innocent Bystanders

**DOI:** 10.1128/mBio.02723-18

**Published:** 2019-01-15

**Authors:** Michael A. Apicella

**Affiliations:** a Department of Microbiology and Immunology, The University of Iowa, Carver College of Medicine, Iowa City, Iowa, USA

**Keywords:** *Neisseria polysaccharea*, antimicrobial resistance, azithromycin, gonorrhea, horizontal gene transfer, hydrophobic efflux pumps, transformation

## Abstract

Neisseria gonorrhoeae is quickly becoming untreatable due to its acquisition of resistance to multiple antimicrobials. It is vital that we begin to understand the mechanisms by which this is occurring. The paper by C. E. Rouquette-Loughlin, J. L. Reimche, J. T. Balthazar, V. Dhulipala, et al. (mBio 9:e02281-18, https://doi.org/10.1128/mBio.02281-18) has shown that horizontal transfer of DNA from a nasopharyngeal commensal, *Neisseria polysaccharea*, has resulted in multiple sequence changes in the *mtr* locus that affect both regulatory and structural regions of the MtrCDE pump, resulting in low-level azithromycin resistance. Studies such as this are increasingly important in our understanding of the movement of resistance between species and for devising strategies to overcome such events.

## Abstract (precis)

Neisseria gonorrhoeae is quickly becoming untreatable due to its acquisition of resistance to multiple antimicrobials. It is vital that we begin to understand the mechanisms by which this is occurring.

## COMMENTARY

Neisseria gonorrhoeae infects between 80 and 100 million individuals yearly (1, 2). Since the 1950s, effective treatment has been based on achieving a cure rate as close to 100% as possible with a single-dose regimen (3). The gonococcus has developed antimicrobial resistance to all drugs previously or currently recommended for single-dose treatment. If new effective single-dose regimens cannot be developed, the possibility of patients developing the preantibiotic-era complications of gonococcal infection is becoming an increasing probability. There are many reasons for this high rate of resistance acquisition, including overuse and misuse of antibiotics, unrestricted access to antibiotics, and suboptimal quality and dosing. Ultimately, the easy facilitation of the gonococcus to acquire extraneous DNA by horizontal gene transfer allows recombination of resistance from other organisms, particularly in the nasopharynx. This site is colonized by many commensal *Neisseria* spp. that can facilitate the emergence and spread of antimicrobial resistance. This is especially true in high-frequency-transmission populations, such as men who have sex with men and commercial sex workers.

The gonococcus is uniquely adapted for horizontal gene transfer. The organism is naturally competent by virtue of its ability to easily move DNA from its external environment into its cytoplasm (1). DNA internalization appears to be a two-step process, in which uptake into the periplasm and transport into the cytoplasm are controlled independently. Among numerous repeat sequences within the gonococcal genome, one 10-bp segment (GCCGTCTGAA) designated the DNA uptake sequence (DUS) occurs approximately once in every 1,100 bp in the genome. N. gonorrhoeae can be inefficiently transformed with non-DUS-containing DNA; however, the efficiency of transformation is increased by orders of magnitude when the incoming DNA contains a DUS, demonstrating a preferential selectivity for DUS-containing DNA. The uptake mechanism has been described as a translocation ratchet mechanism (4). This process is based on the ability of DNA to passively move through the PilQ pore, perhaps with the assistance of pilus retraction, and is considered to bind to ComE via the 10-bp DNA uptake sequence. DNA accumulates in the periplasmic space and is moved into the cytoplasm by a second transporter, possibly ComA. While the DNA of commensal *Neisseria* organisms do not contain a high frequency of the DUS repeats (1), the high DNA homology which occurs between these closely related species allows homologous-recombination events to occur at a relatively high frequency.

For this reason, a broad-based genomic approach is necessary to improve our understanding of the factors involved in the emergence of antimicrobial resistance, the mechanisms of resistance, and its transmission in gonococcal strains.

The *mtr* locus encodes a pump which captures and exports structurally diverse, but generally amphipathic, antimicrobial agents, including macrolides, beta-lactams, cationic antimicrobial peptides, dyes, and detergents (5). It is composed of multiple genes encoding structural (MtrCDE) and regulatory (MtrR and MtrA) proteins. The contribution of this efflux pump in antimicrobial resistance expressed by gonococci can be enhanced by *cis*- or *trans*-acting mutations that result in overexpression of the *mtrCDE* efflux pump operon. Importantly, overproduction of the MtrCDE efflux pump due to relief of transcriptional repression of *mtrCDE* can contribute to clinically relevant levels of resistance to beta-lactams and macrolides. Previous studies by Wadsworth et al. indicate that the *mtr* locus is a hot spot in the gonococcal genome for recombination and is susceptible to frequent transformative events (6). The studies of the *mtr* locus in azithromycin-resistant gonococci, which are described in the *mBio* paper by Rouquette-Loughlin et al. (7), give a detailed analysis of such a series of events.

These authors studied eight N. gonorrhoeae strains with low-level resistance to azithromycin in which complete genome sequencing indicated the absence of a 23S rRNA mutation associated with high-level azithromycin resistance. Sequence analysis of the *mtr* loci of the eight strains indicated that the sequences were identical and that they differed at multiple nucleotide sites from a well-studied azithromycin-sensitive gonococcal strain, FA19. Their studies demonstrated that the *mtr* locus from one of the resistant strains was almost identical (95% identity) to the *mtr* locus of a nasopharyngeal commensal, Neisseria polysaccharea. Using phylogenetic tree analysis of the *mtr* locus, they demonstrated that gonococcal strains acquired the *mtr* locus mutations by horizontal gene transfer from commensal *Neisseria* organisms. The authors showed that significant decreased expression of *mtrR* and increased expression of *mtrE* occurred with the eight mutants studied compared to the levels of expression in the sensitive FA19 strain. In a series of carefully documented experiments, the investigators showed that single nucleotide changes in overlapping regions of the divergently expressed promoters of *mtrR* and *mtrCDE* (2), as well as amino acid changes in the antibiotic binding region of the terminal peptide in the MtrD transporter, led to the similar increased resistance levels seen in the azithromycin-resistant strains.

This paper is a very complete study which has carefully delineated the mutations in a group of gonococci with low-level resistance to azithromycin and shown the results of such mutations with carefully performed functional studies in a previously sensitive gonococcal strain. The transmission of resistance from a nasopharyngeal source to the gonococcus has been shown with movement of mosaic *penA* sequence changes, but this is the first time a similar effect has been shown with another antimicrobial agent. The results emphasize that changes both in the transcription of regulatory genes and in amino acids in structural genes can result from the horizontal transfer of DNA. This paper also demonstrates that the association of the gonococcus with a commensal bacterium at an extragenital site can result in the transformation of previously sensitive gonococci to resistant gonococci.

The Rouquette-Loughlin et al. (7) paper and the previous *mBio* paper by Wadsworth et al. (6) point out the importance of better understanding the relationship between related commensals and pathogens at sites where the two comingle. Both papers describe the analysis of horizontal spread of antimicrobial resistance from a commensal organism sharing a common environment with a pathogen. One has delineated the interchange between the commensal and pathogen, and the other has detailed the exact nature of the lesions, confirming their role in the resistance process. These papers can serve as an example of what is necessary to get a firmer understanding of the participants and the mechanisms which allow antibiotic resistance to move from commensal to pathogen.
